# Supplementary figures and images for: Applications of machine learning in decision analysis for dose management for dofetilide
Source: PLoS One. 2019 Dec 31;14(12):e0227324. doi: 10.1371/journal.pone.0227324 (PMC6938356; doi:10.1371/journal.pone.0227324)

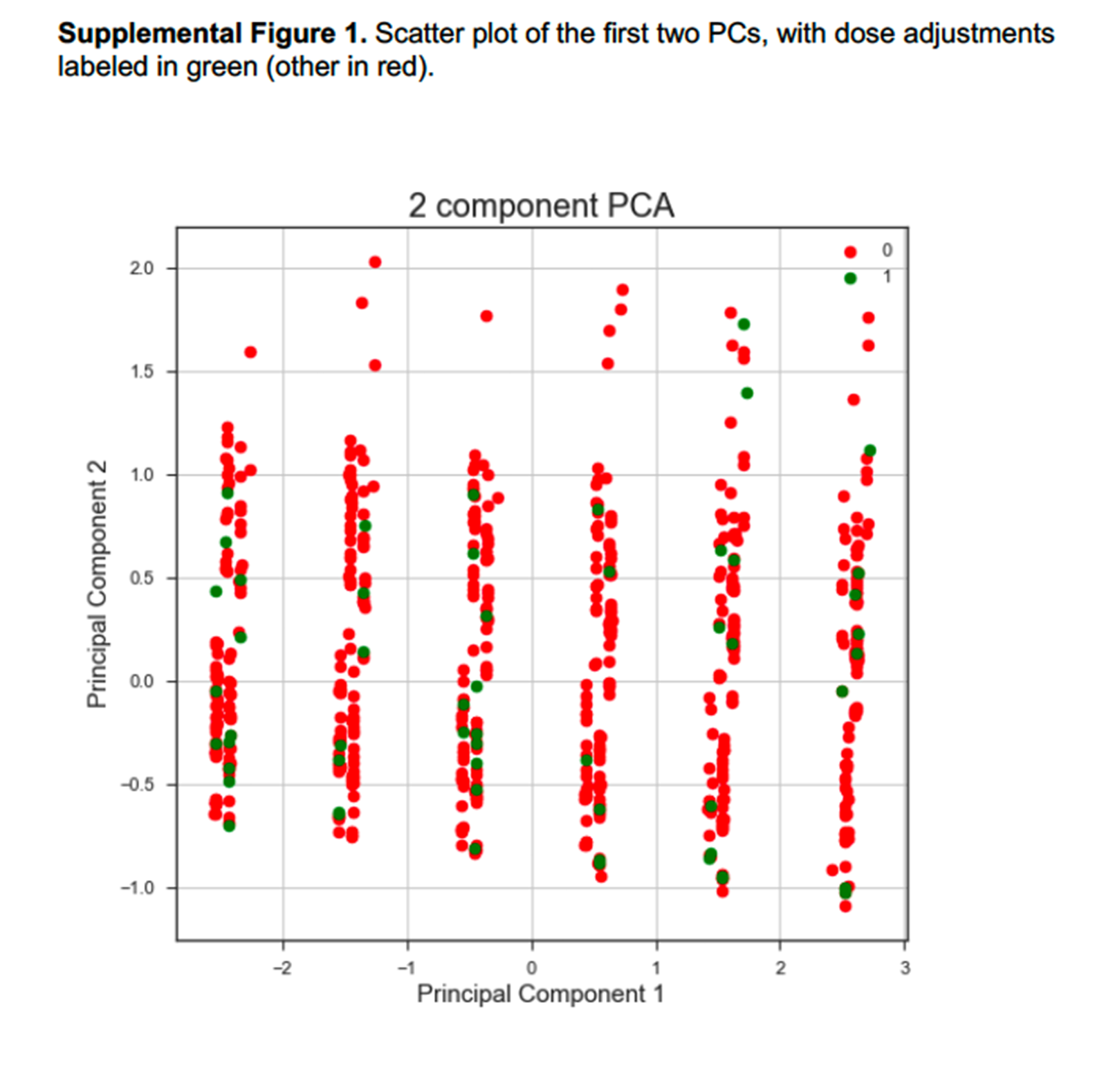

Supplement: S1 Fig — (TIF) [file pone.0227324.s001.tif]

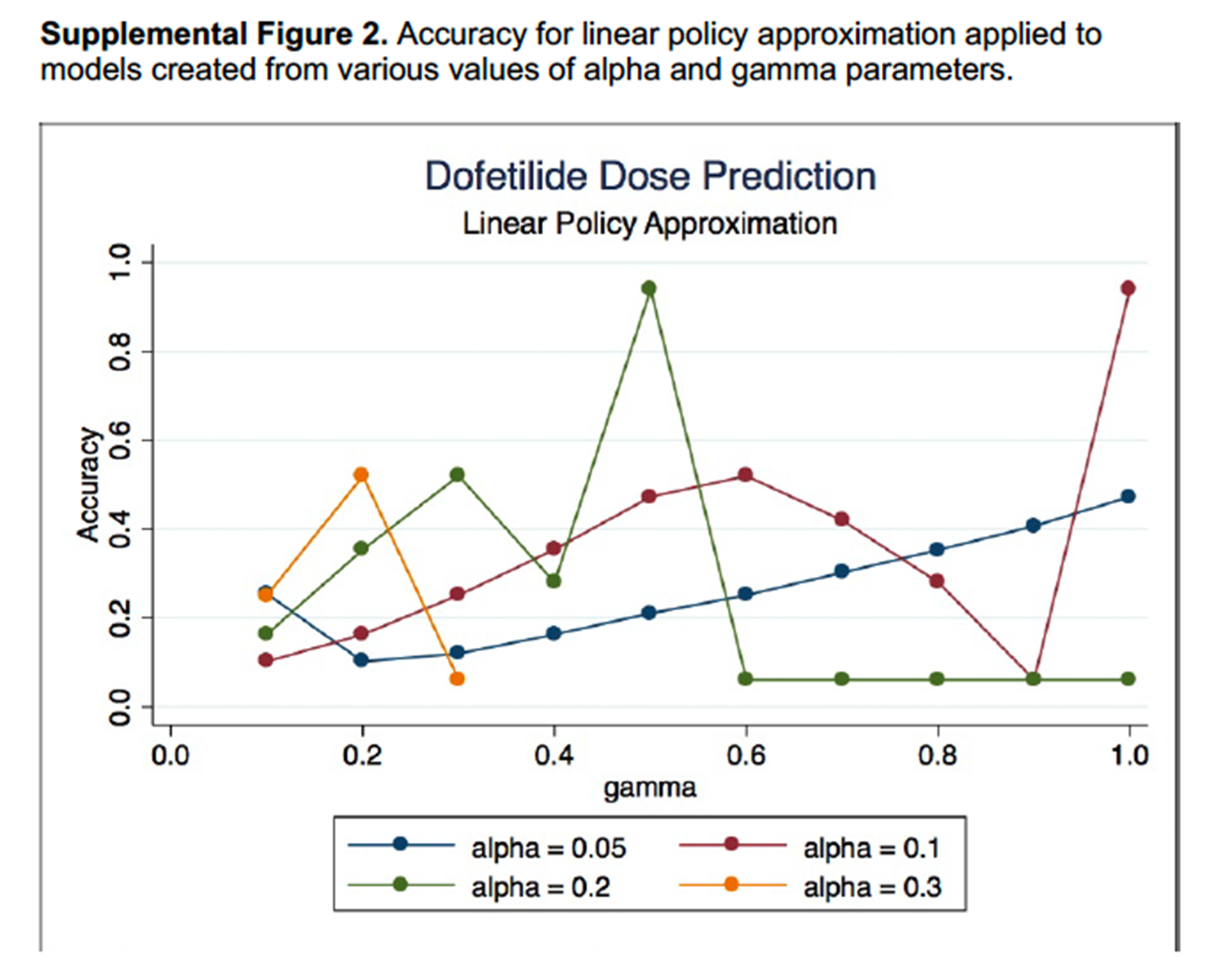

Supplement: S2 Fig — (TIF) [file pone.0227324.s002.tif]
