# Supplementary material for: Applications of machine learning in decision analysis for dose management for dofetilide
Source: PLoS One. 2019 Dec 31;14(12):e0227324. doi: 10.1371/journal.pone.0227324 (PMC6938356; doi:10.1371/journal.pone.0227324)
Supplement: S1 Supplemental Methods — (DOCX) [file pone.0227324.s003.docx]

**Supplemental Material**

**Supplemental Methods**

Packages used for analysis include the following:

Unsupervised Learning:

- *sklearn.decomposition.PCA* for principal component analysis
- *sklearn.cluster.KMeans* for K-means clustering with 8 clusters.

Supervised Learning:

- *sklearn.linear_model.LogisticRegression* for L1 regularized logistic regression algorithm with ‘liblinear’ solver
- *sklearn.ensemble.RandomForestClassifier* for Random Forest classification with 500 estimators, and maximum leaf nodes of 20
- *sklearn.ensemble.AdaBoostClassifier* for Boosted decision tree classification, combined with
- *sklearn.tree.DecisionTreeClassifier* with 200 estimators, SAMME.R algorithm, and learning rate of 0.5
- *sklearn.svm.SVC* for support vector machine classification with radial basis function kernel, gamma of 5, and C-value of 0.001
- *sklearn.neighbors.KNeighborsClassifier* for K-nearest neighbors classification with 1 and 10 nearest neighbors
