# Supplementary material for: Applications of machine learning in decision analysis for dose management for dofetilide
Source: PLoS One. 2019 Dec 31;14(12):e0227324. doi: 10.1371/journal.pone.0227324 (PMC6938356; doi:10.1371/journal.pone.0227324)
Supplement: S1 Table — (DOCX) [file pone.0227324.s004.docx]

**Supplemental Table 1A. Cluster characteristics for k = 4 clusters**

| **Cluster** | **1** | **2** | **3** | **4** |
| --- | --- | --- | --- | --- |
| **Number** | 307 | 384 | 657 | 703 |
| **Dose position (% of dose)** | 2-5 (1.6%)  3-139 (45.3%)  4-163 (53.1%) | 3-202 (52.6%)  4-182 (47.4%) | *5-334 (50.8%)*  6-323 (49.2%) | 1-357 (50.8%)  2-346 (49.2%) |
| **Dose amount** | 125mcg: 43 (14.0%)  250mcg: 239 (77.9%)  Missing/None: 25 (8.1%) | 500mcg: 384 (100%) | 125mcg: 55 (8.4%)  250mcg: 219 (33.3%)  500mcg: 287 (43.7%)  Missing/None: 96 (14.6%) | 125mcg: 16 (2.3%)  250mcg: 194 (27.6%)  500mcg: 479 (68.1%)  Missing/None: 14 (2.0%) |
| **Age (years)** | 69.3 ± 10.9 | 64.5 ± 10.0 | 66.7 ± 10.6 | 66.6 ± 10.6 |
| **Female Sex** | 127 (41.4%) | 84 (21.9%) | 200 (30.4%) | 215 (30.6%) |
| **Sinus Rhythm** | 169 (55.1%) | 228 (59.4%) | 514 (78.2%) | 279 (39.7%) |
| **Heart rate (bpm)** | 72.6 ± 18.2 | 70.2 ± 16.0 | 68.2 ± 16.1 | 78.3 ± 19.6 |
| **QRS (ms)** | 103.1 ± 30.4 | 102.6 ± 23.8 | 102.4 ± 24.4 | 102.8 ± 28.6 |
| **QTc (ms)** | 485.2 ± 40.9 | 465.8 ± 35.5 | 473.5 ± 37.6 | 457.2 ± 41.1 |
| **Creatinine (mg/dL)** | 1.07 ± 0.29 | 0.98 ± 0.22 | 1.02 ± 0.26 | 1.01 ± 0.25 |
| **Beta Blocker** | 193 (62.9%) | 209 (54.4%) | 380 (57.8%) | 402 (57.2%) |
| **CCB** | 75 (24.4%) | 87 (22.7%) | 149 (22.7%) | 165 (23.5%) |
| **CHF** | 50 (16.3%) | 35 (9.1%) | 78 (11.9%) | 83 (11.8%) |
| **CAD** | 84 (27.4%) | 61 (15.9%) | 135 (20.6%) | 143 (20.3%) |
| **HTN** | 149 (48.5%) | 162 (42.2%) | 303 (46.1%) | 311 (44.2%) |
| **DM** | 33 (10.8%) | 46 (12.0%) | 77 (11.7%) | 83 (11.8%) |
| **PPM** | 20 (6.5%) | 25 (6.5%) | 45 (6.9%) | 41 (5.8%) |
| **ICD** | 25 (8.1%) | 19 (5.0%) | 38 (5.8%) | 46 (6.5%) |
| **LVEF (%)** | 54.3 ± 12.5 | 54.2 ± 13.0 | 54.5 ± 12.5 | 54.3 ± 12.9 |

All values listed at mean ± SD or number (%). Sinus rhythm = sinus or atrial paced rhythm (not atrial fibrillation/flutter); CCB = Calcium channel blocker; CHF = heart failure; CAD = coronary artery disease; HTN = hypertension; DM = diabetes mellitus; PPM = pacemaker present; ICD = implantable cardioverter-defibrillator present; LVEF = left ventricular ejection fraction based on transthoracic echocardiography

**Supplemental Table 1B. Q table for k = 4 clusters**

| **Cluster** | **Keep Dose** | **Lower Dose** |
| --- | --- | --- |
| **1** | -0.00017 | -1.6e-05 |
| **2** | -0.0049 | 0.0 |
| **3** | -0.23 | -0.12 |
| **4** | -0.0070 | -7.3e-05 |
