# Supplementary material for: Applications of machine learning in decision analysis for dose management for dofetilide
Source: PLoS One. 2019 Dec 31;14(12):e0227324. doi: 10.1371/journal.pone.0227324 (PMC6938356; doi:10.1371/journal.pone.0227324)
Supplement: S2 Table — (DOCX) [file pone.0227324.s005.docx]

**Supplemental Table 2A. Cluster characteristics for k = 6 clusters**

| **Cluster** | **1** | **2** | **3** | **4** | **5** | **6** |
| --- | --- | --- | --- | --- | --- | --- |
| **Number** | 384 | 274 | 202 | 287 | 331 | 573 |
| **Dose position (% of dose)** | 3 – 202 (52.6%)  4 – 182 (47.4%) | 1 – 8 (2.9%)  2 – 127 (46.4%)  3 – 139 (50.7%) | 6 – 202 (100%) | 5 – 166 (57.8%)  6 – 121 (42.2%) | 4 – 163 (49.2%)  5 – 168 (50.8%) | 1 – 349 (60.9%)  2 – 224 (39.1%) |
| **Dose amount** | 500mcg: 384 (100%) | 125mcg: 39 (14.2%)  250mcg: 78.5%  Missing/none: 20 (7.3%) | 125mcg: 24 (11.9%)  250mcg: 96 (47.5%)  Missing/none: 82 (40.6%) | 500mcg: 287 (100%) | 125mcg: 51 (15.4%)  250mcg: 251 (75.8%)  Missing/none: 29 (8.8%) | 250mcg: 90 (15.7%  500mcg: 479 (83.6%)  Missing/none: 4 (0.7%) |
| **Age (years)** | 64.5 ± 10.0 | 69.5 ± 11.1 | 67.9 ± 10.7 | 64.9 ± 9.8 | 68.6 ± 11.0 | 65.9 ± 10.2 |
| **Female Sex** | 84 (21.9% | 115 (42.0%) | 72 (35.6%) | 61 (21.3% | 133 (40.2%) | 161 (28.1%) |
| **Sinus Rhythm** | 228 (59.4%) | 122 (44.5%) | 172 (85.2%) | 229 (79.8%) | 210 (63.4%) | 229 (40.0%) |
| **Heart rate (bpm)** | 70.2 ± 16.0 | 75.0 ± 18.9 | 68.3 ± 15.9 | 65.9 ± 13.6 | 71.8 ± 18.6 | 78.7 ± 19.7 |
| **QRS** | 102.6 ± 23.8 | 103.9 ± 30.6 | 101.3 ± 24.1 | 104.3 ± 24.8 | 101.3 ± 25.0 | 102.7 ± 29.2 |
| **QTc** | 465.8 ± 35.5 | 483.0 ± 46.4 | 475.4 ± 36.5 | 468.6 ± 35.2 | 481.5 ± 40.6 | 452.4 ± 37.2 |
| **Creatinine** | 0.98 ± 0.22 | 1.08 ± 0.30 | 1.03 ± 0.27 | 0.98 ± 0.23 | 1.06 ± 0.28 | 0.99 ± 0.24 |
| **Beta Blocker** | 209 (54.4%) | 173 (63.1%) | 117 (57.9%) | 162 (56.5%) | 202 (61.0%) | 321 (56.0%) |
| **CCB** | 87 (22.7%) | 68 (24.8%) | 50 (24.8%) | 59 (20.6%) | 81 (24.5%) | 131 (22.9%) |
| **CHF** | 35 (9.1%) | 45 (16.4%) | 29 (14.4%) | 24 (8.4%) | 52 (15.7%) | 61 (10/7%) |
| **CAD** | 61 (15.9%) | 71 (25.9%) | 49 (24.3%) | 47 (16.4%) | 83 (25.1%) | 112 (19.6%) |
| **HTN** | 162 (42.2%) | 126 (46.0%) | 100 (49.5%) | 121 42.2%) | 163 (49.2%) | 253 (44.2%) |
| **DM** | 46 (12.0%) | 27 (9.9%) | 23 (11.4%) | 35 (12.2%) | 37 (11.1%) | 71 (12.4%) |
| **PPM** | 25 (6.5%) | 15 (5.5%) | 15 (7.4%) | 20 (7.0%) | 21 (6.3%) | 35 (6.1%) |
| **ICD** | 19 (5.0%) | 23 (8.4%) | 12 (5.9%) | 16 (5.6%) | 23 (7.0%) | 35 (6.1%) |
| **LVEF** | 54.2 ± 13.0 | 54.4 ± 13.0 | 54.9 ± 11.8 | 54.4 ± 13.0 | 54.1 ± 12.6 | 54.3 ± 12.7 |

All values listed at mean ± SD or number (%). Sinus rhythm = sinus or atrial paced rhythm (not atrial fibrillation/flutter); CCB = Calcium channel blocker; CHF = heart failure; CAD = coronary artery disease; HTN = hypertension; DM = diabetes mellitus; PPM = pacemaker present; ICD = implantable cardioverter-defibrillator present; LVEF = left ventricular ejection fraction based on transthoracic echocardiography

**Supplemental Table 2B. Q table for k = 6 clusters**

| **Cluster** | **Keep Dose** | **Lower Dose** |
| --- | --- | --- |
| **1** | -0.054 | -0.00052 |
| **2** | -0.00080 | -0.00014 |
| **3** | -0.37 | -5.90 |
| **4** | -0.85 | -7.4e-05 |
| **5** | -0.00013 | -6.9e-05 |
| **6** | -0.038 | -0.0015 |
