# Supplementary material for: Applications of machine learning in decision analysis for dose management for dofetilide
Source: PLoS One. 2019 Dec 31;14(12):e0227324. doi: 10.1371/journal.pone.0227324 (PMC6938356; doi:10.1371/journal.pone.0227324)
Supplement: S3 Table — (DOCX) [file pone.0227324.s006.docx]

| **Parameter** | **No change** | **Dose change** |
| --- | --- | --- |
| age_years | 7.56E+185 | 1028.198506 |
| bmi | 3.25E+185 | -8.365964 |
| hr_ | 5.38E+185 | -1161.59328 |
| qtc_cpu_based_ | 4.57E+185 | -941.241576 |
| tte_lvef | 6.79E+185 | -848.112759 |
| potassium | 5.30E+185 | 276.983848 |
| magnesium | 4.46E+185 | -170.593196 |
| creatinine | 2.48E+185 | 721.813547 |
| betablocker | -1.01E+185 | 1995.601825 |
| ccb | -2.79E+185 | -640.978418 |
| qrs_duration_ | 3.95E+184 | -144.240687 |
| sex | 3.99E+185 | -189.105307 |
| indication | 3.35E+74 | -0.315224 |
| SR_ | 3.07E+185 | 844.658958 |
| ppm | 6.01E+185 | 2169.177153 |
| CV_ | 1.44E+185 | 1733.051941 |
| dose_ | 9.93E+185 | -3406.121237 |
| htn | 2.95E+182 | 102.409849 |
| icd | 9.86E+185 | -457.81708 |
| afib | -7.61E+176 | 279.38327 |
| vt | 9.87E+185 | -861.85385 |
| dm | 6.01E+185 | -277.424116 |
| cad | 5.87E+185 | -1707.796601 |
| chf | 9.86E+185 | -2100.37959 |
| dose | 3.23E+185 | 373.687832 |

**Supplemental Table 3. Weights for most accurate predictive model.**  Alpha = 0.1, gamma = 1.0; accuracy = 0.939.
